# Dynamics of co-authorship and productivity across different fields of scientific research

**DOI:** 10.1371/journal.pone.0189742

**Published:** 2018-01-10

**Authors:** Austin J. Parish, Kevin W. Boyack, John P. A. Ioannidis

**Affiliations:** 1 Meta-Research Innovation Center at Stanford (METRICS), Stanford University, Stanford, California, United States of America; 2 SciTech Strategies, Inc., Albuquerque, New Mexico, United States of America; 3 Meta-Research Innovation Center at Stanford (METRICS), Stanford University, Stanford, California, United States of America; 4 Stanford Prevention Research Center, Department of Medicine, Stanford University School of Medicine, Stanford, California United States of America; 5 Department of Health Research and Policy, Stanford University School of Medicine, Stanford, California, United States of America; 6 Department of Biomedical Data Science, Stanford University School of Medicine, Stanford, California; 7 Department of Statistics, Stanford University School of Humanities and Sciences, Stanford, California, United States of America; KU Leuven, BELGIUM

## Abstract

We aimed to assess which factors correlate with collaborative behavior and whether such behavior associates with scientific impact (citations and becoming a principal investigator). We used the R index which is defined for each author as log(N_p_)/log(I_1_), where I_1_ is the number of co-authors who appear in at least I_1_ papers written by that author and N_p_ are his/her total papers. Higher R means lower collaborative behavior, i.e. not working much with others, or not collaborating repeatedly with the same co-authors. Across 249,054 researchers who had published ≥30 papers in 2000–2015 but had not published anything before 2000, R varied across scientific fields. Lower values of R (more collaboration) were seen in physics, medicine, infectious disease and brain sciences and higher values of R were seen for social science, computer science and engineering. Among the 9,314 most productive researchers already reaching N_p_ ≥ 30 and I_1_ ≥ 4 by the end of 2006, R mostly remained stable for most fields from 2006 to 2015 with small increases seen in physics, chemistry, and medicine. Both US-based authorship and male gender were associated with higher values of R (lower collaboration), although the effect was small. Lower values of R (more collaboration) were associated with higher citation impact (h-index), and the effect was stronger in certain fields (physics, medicine, engineering, health sciences) than in others (brain sciences, computer science, infectious disease, chemistry). Finally, for a subset of 400 U.S. researchers in medicine, infectious disease and brain sciences, higher R (lower collaboration) was associated with a higher chance of being a principal investigator by 2016. Our analysis maps the patterns and evolution of collaborative behavior across scientific disciplines.

## Introduction

Collaboration is now seen as essential to progress in scientific research, and over the past several decades large-scale collaborative projects have become increasingly frequent in fields as diverse as medicine, genetics, and high-energy physics [1–3]. Although these large collaborations have received more media attention [4], collaboration on a smaller scale is also important for scientific productivity [5].

The average number of co-authors per paper published by individual scientists has steadily increased in all fields over the past century [6]. The possible effect of collaboration on improving scientific efficiency and productivity is particularly appealing. Governments and research institutions have been trying for many years to increase "research collaboration", either to increase the advancement of knowledge or to increase the efficiency/effectiveness of research [5]. Additionally, inspired by the possible effects on scientific productivity and the expected benefits of encouraging collaboration, universities have developed research centers with this goal in mind [7].

Increased collaboration has long been found to be associated with increased scientific productivity using individual researchers as the unit of study [8–13]. Collaboration is also frequently mentioned as an important factor in scientists’ own reflections on their success [14].

A variety of approaches have been taken to study the relationship between collaboration and scientific success. Researchers have examined the relationship between features of small scientific teams and their collective research output [15], finding that team cohesion and disciplinary diversity is associated with higher productivity. In analyses looking at papers as the unit of study, increased co-authorship has been associated with citation impact [16]. Within the field of information systems, for instance, total number of co-authors of a study is positively correlated with the number of citations received by that study [17]. This positive effect of total number of co-authors on citations has also been found across scientific fields in Italy [18], in the field of demography [19], as well as in Russian molecular biologists [20].

Many studies have also examined collaboration in the context of co-authorship networks, using metrics such as degree centrality [21,22], eigenvector centrality [23] and betweenness centrality [24,25]. Networks metrics such as these have been found to be associated with scientific productivity in a variety of scientific fields, including informetrics [26], forest management [22], nanoscience, pharmacology and statistics [21]. However, the effect of collaboration dynamics on productivity may vary across scientific fields. For instance, Jansen et al found that in the newer field of nanoscience, more researchers in “gatekeeper” roles, linking together disconnected groups of researchers, was associated with greater research output; they found the opposite effect in astrophysics [27]. Given that most studies have examined these patterns in specific scientific disciplines (one or a few at a time), it would be useful to assess systematically to what extent such relationships vary when all scientific disciplines are considered.

A researcher’s productivity may also shape their *future* role in networks of co-authors, with greater scientific success and exposure allowing the researcher more opportunities to collaborate. For example, in a set of Canadian researchers past productivity was associated with increased eigenvector centrality [23]. Indeed, the correlation between collaboration and productivity may become stronger as a researcher progresses in his or her career [28]. Later career scientists tend to engage in collaboration more frequently, both within their disciplines and in interdisciplinary research [29].

Highly collaborative authors also seem to cite more recently published articles and to re-cite (citing the same references in multiple papers) less frequently, and thus may dwell closer to and push the frontiers of research [30]. International collaboration in particular seems to be strongly related to productivity, as measured by total publications [31]. However, it is unclear whether authors who collaborate a lot can also be principal investigators leading their own research agenda. It is unknown whether collaborative and leadership behavior may compete against each other.

The number of ties that researchers form as they collaborate may not have as large of an effect as the nature of those ties. For example, physicists who form persistent ties, collaborating repeatedly with stable groups of co-authors, seemed to have increased chances of publishing in high ranking journals [32]. Persistent ties (defined by repeated association and collaboration) had greater effect on rate of research productivity than new ties did [33]. Additionally, Bordons et al found that for Spanish researchers from the fields of nanoscience, pharmacology and statistics, both tie strength and degree centrality were associated with an author’s scientific productivity as measured by the g-index [21]. Therefore, in evaluating the impact of collaborations, one needs to use metrics that take also into account whether collaboration is with the same people or with different researchers each time.

In this study, we analyze collaborative dynamics on a large scale, across 249,054 researchers collected from data from the Scopus database (https://www.scopus.com/) for the period 2006–2015. Using a previously proposed index for estimating the collaborative behavior of individual researchers [1] we examine how collaboration varies across scientific fields, how this behavior is changing over time in different fields, and what individual factors are associated with collaborative behavior. We also investigate how collaborative behavior influences citation impact, and whether this behavior can predict principal investigator (PI) status for a scientist in the future. Our analysis aims to offer comparative evidence on the pattern of collaborative behavior, its evolution over time and its potential relationships with impact and scientific leadership across all disciplines of science.

## Materials and methods

### Measuring network-adjusted researcher collaboration

In this work, we use the metrics for collaboration previously proposed by Ioannidis [1], measuring co-authorship for an individual researcher primarily in terms of two quantities, I_1_ and R. I_1_ is formulated in an h-index like fashion–for a given researcher, I_1_ is the number of authors who appear in at least I_1_ papers with that researcher. For example, if a researcher has written 8 papers with a consistent set of 7 other co-authors, that researcher's I_1_ will be equal to 7.

With more publications there are more opportunities for co-authorship and for more papers written in common with each co-author. Thus, we expect I_1_ to rise as a function of the total number of publications, N_p_ [1]. Assuming a power law relationship, we can write N_p_ = (I_1_)^R^, and in turn obtain R = log(N_p_) / log(I_1_).

Importantly, a lower value of R implies a more collaborative author, while a larger value of R implies a less collaborative author. When an author writes more papers with new, frequently changing sets of co-authors, these changing sets will not contribute much to I_1_ despite N_p_ increasing. If an author works with very few co-authors per paper, for the same N_p_ the author’s I_1_ value would be smaller and in turn his or her R value would be larger. Thus, high values of R appear both in those who simply rarely work with others, or work with others but don’t collaborate repeatedly with the same co-authors [1]. In line with results of previous research which has shown that persistent, repeated co-authorship is strongly associated with productivity [21,32,33], we operationalize “collaboration” in this way to refer to working again and again with stable groups of co-authors, and R efficiently captures this notion.

### Sources of data

Information was extracted for all authors from the SCOPUS database in April 2016. Data was restricted to SCOPUS authors with at least 30 publications and who were still publishing in 2015, but who did not publish anything before 2000. This original extracted dataset contained 249,054 authors. A variety of information was then extracted or calculated for these authors using the data available in SCOPUS. The metrics that were obtained for each researcher include that researcher’s h-index for citations [34], total number of publications, total number of co-authors, R index, research field, first year of publishing activity, main country that the author publishes in, and the author’s gender. For the h-index, number of publications, number of co-authors, and R-index, these metrics were obtained for years 2000, 2003, 2006, 2009, 2012, and 2015. Additionally, for a randomly selected subset of 400 U.S. based researchers from the fields of medicine, brain sciences, and infectious diseases, Federal Reporter (https://federalreporter.nih.gov/) was used to determine if that researcher was a PI on a federally funded project as of April 2016. All original data associated with this work can be found online via the Open Science Framework repository at https://osf.io/bptgy/.

The predicted research field for each author was obtained using methods and data developed by Boyack and Klavans [35]. Using their algorithm, authors were assigned to one of the following 12 fields: computer science, physics, chemistry, engineering, earth sciences, biology, infectious disease, medicine, health sciences, brain research, social sciences, or the humanities.

Larivière et [36] developed an accurate method of identifying the gender of a researcher using the researcher’s name, through a manually validated master list of name-gender assignments. This database and code was used in this work to assign gender, by courtesy of Larivière et al. Their method is overall accurate, but its gender assignation rates vary by country. For instance, the United States, Germany, the United Kingdom, Italy and Australia all have greater than 90% gender assignation success, but much lower rates (between 60% and 70%) are found for India, South Korea, Taiwan, or the Netherlands.

Of the original 249,054 authors, 3201 (1.3%) did not have a defined R value in any year. These were researchers who never co-authored twice with the same person, thus having an I_1_ value of 1 and an indeterminate value of R. In all presented analyses, these 3201 researchers are removed from the dataset. After removing these researchers, as well as researchers who could not be assigned a field and 45 researchers who were assigned the field “Humanities” (too few to meaningfully analyze), the final size of the overall dataset was 245,543 researchers.

When the values of either N_p_ or I_1_ are very small, small changes in them may result in large changes in R, rendering it an imprecise measure. With this in mind, for tracking changes in R over time we used a set of authors with a total number of publications greater than or equal to 30, and a value of I_1_ greater than or equal to 4 by the end of 2006. This resulted in a set of 9,314 authors. To determine which factors are associated with R, we restrict our analysis to individuals with Np ≥ 30 and I1 ≥ 4 in 2015 (yielding a set of 233,798 researchers), those who were identified as "male" or "female" by the algorithm mentioned above, excluding those defined as "unknown" (yielding 113,172 researchers), and finally only including the subset of authors who were given a defined country, yielding a total of 92,558 individuals [36].

**Fig 1:**
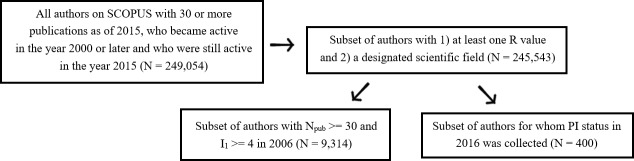
Data collection flowchart for this study. “N_pub_” is the total number of publications of that author.

Finally, a set of 400 U.S. based authors working in the fields of medicine, brain or infectious disease were randomly selected from this set of 245,543. For these 400 selected authors, an additional datum, whether the author was a PI on a U.S. federally funded research grant (funded by any of NIH, NSF, NASA, EPA, DOD, AHRQ, FDA, CDC, VA or others) by April 2016, was collected. PI status was determined by perusing the Research Portfolio Online Reporting Tools site maintained by the National Institutes of Health (NIH) (https://report.nih.gov/). The process of data collection is shown in Fig 1 below.

### Procedures

The median and range of I_1_ and R was obtained for each field and overall, and the change in R over time was visualized for each field. The association between R and first year of publication, total number of publications by 2015, country of origin, gender and field was assessed using linear regression. Additionally, the association between R and scientific citation impact as measured by the h-index [34] was studied. Finally, for the subset of 400 U.S. based authors, the association between R and PI status was studied with linear regression. All statistical processing was done using the R Statistical Programming Language [37].

## Results

### General features of the distribution of R and I

The number of authors from each field were as follows: biology, 15,659 (6.3%); brain sciences, 11,990 (4.8%); chemistry, 25,711 (10.3%); computer science, 40,389 (16.2%); earth science, 5,274 (2.1%); engineering, 26,509 (10.6%); health sciences, 13,664 (5.5%); humanities, 45 (0.02%); infectious disease, 7,874 (3.2%); medicine, 55843 (22.4%); physics, 37,229 (14.9%); social sciences, 7,555 (3.0%); unable to assign, 1,312 (0.5%). Three fields together accounted for over half of the researchers: medicine (22.4%), computer science (16.2%) and physics (15.0%). Table 1 below shows the median I_1_ and R values, as well as the quantiles of R, across the fields considered.

**Table 1:** Median of I_1_ values, and median/percentiles of R values in 2015 across all researchers, separated by research field. This data was obtained using the full set of 245,543 authors.

Field	I_1_ median	R median	R20	R40	R60	R80
Biology	6	2.19	1.98	2.13	2.26	2.50
Brain Sciences	6	2.06	1.84	1.98	2.13	2.34
Chemistry	6	2.14	1.94	2.09	2.19	2.39
Computer Science	5	2.28	2.03	2.20	2.37	2.57
Earth Sciences	6	2.18	1.93	2.11	2.26	2.50
Engineering	5	2.24	2.04	2.18	2.33	2.54
Health Sciences	6	2.17	1.94	2.11	2.24	2.50
Infectious Disease	6	2.05	1.85	1.98	2.12	2.31
Medicine	7	2.00	1.79	1.94	2.09	2.26
Physics	9	1.80	1.05	1.64	1.92	2.19
Social Sciences	4	2.61	2.28	2.52	2.76	3.23
***All Fields***	6	2.13	1.86	2.04	2.20	2.46

The value of R varies across fields. Fields with significantly more collaboration than average, as measured by R, include physics, medicine, infectious disease, and brain sciences; less collaborative fields include social sciences, computer science, and engineering. An analysis of variance (ANOVA) model of R versus field yielded an F-statistic of 7.9 x 10^3^ (p<0.0001), indicating significant heterogeneity across fields. The values of R also range widely within each field. The lower 20% ranged from 1.05 to 2.28 across fields and the higher 20% (80^th^ percentile) ranged from 2.19 to 3.23 across fields.

The lowest median R values (highest collaborative behavior) were seen in physics. In fact, in physics, some authors have R values of 1.0, implying the existence of authors whose I_1_ value is equal to their total number of publications (in 2015). Indeed, there are a total of 1703 such physicists (4.6% of all physicists included in this sample), with a total publication count between 30 and 403 and a median publication count of 80. Many more physicists have values very close to 1, and a full 20.0% have values less than 1.05.

### R over time by field

**Fig 2:**
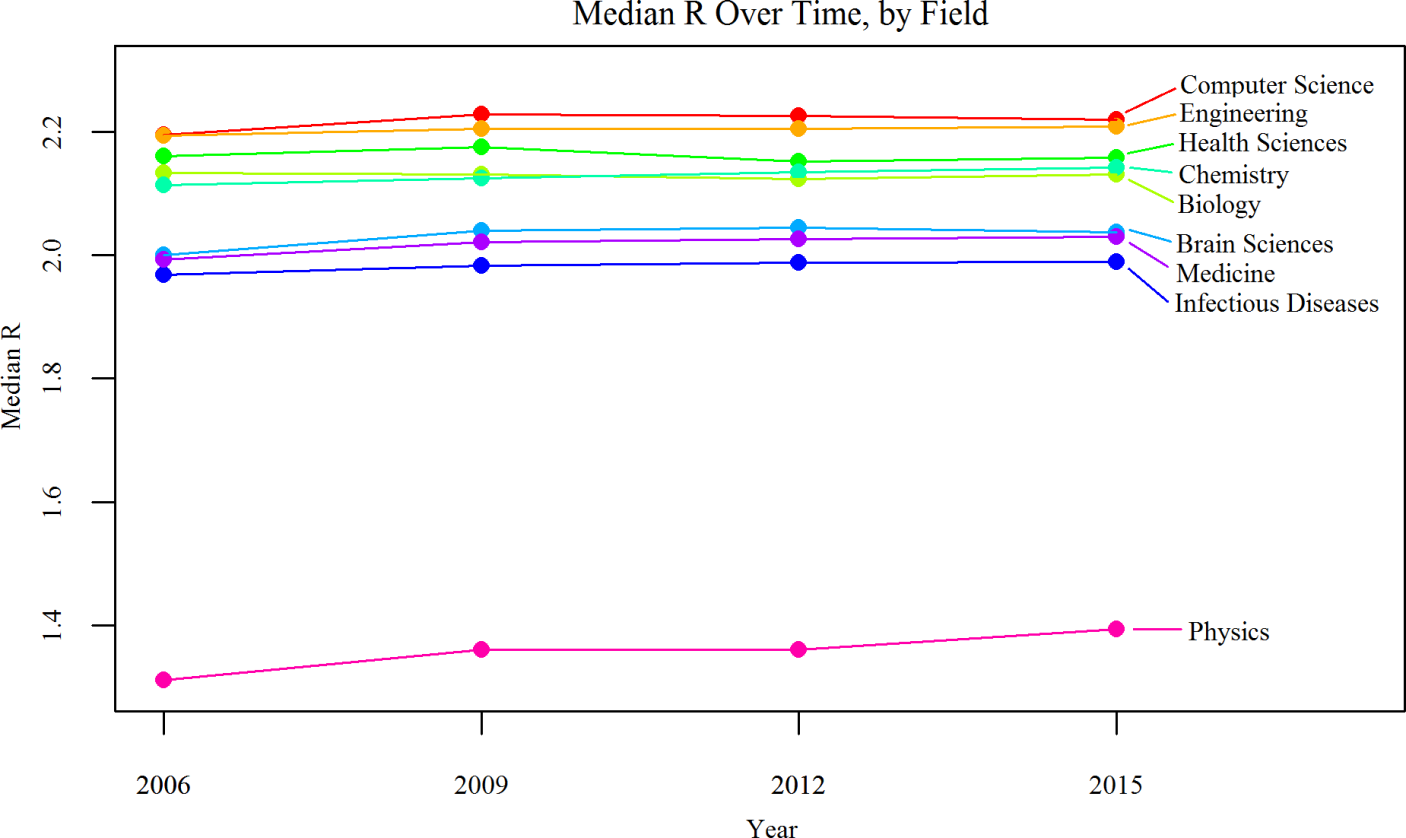
Median R value over time for each field, for the set of 9,314 researchers with 30 or more publications and an I_1_ of 4 or more by the end of 2006. Data is shown for fields with 100 or more authors.

To visualize the change in R over time, the median R value was obtained for each field in every year and the results were plotted from 2006 to 2015. We restricted this analysis to the subset of 9,314 researchers whose N_p_ was ≥ 30 and whose I_1_ was ≥ 4 by the end of 2006, that is, authors who had already shown to be prolific enough to allow for a meaningful assessment of their further trajectory over 3-year intervals. The results are shown in Fig 2 below. They suggest that the median R remains rather constant within each scientific field with relatively little fluctuation or change in the relative ranking of different fields. The field with the smallest R (physics) shows a small increase over time (0.0041 per year, p<0.001) and statistically significant (but even smaller in magnitude) increases are documented in chemistry and medicine, while no statistically significant changes are seen in the other fields. Across all fields, there is a very small increase in R (0.0020 per year, p<0.001) over time.

### Correlates of R

To determine which factors are associated with R, we restrict our analysis to individuals with N_p_ ≥ 30 and I_1_ ≥ 4 in 2015 (yielding a set of 233,798 researchers), those who were identified as "male" or "female" by the algorithm mentioned above, excluding those defined as "unknown" (yielding 113,172 researchers), and finally only including the subset of authors who were given a defined country, yielding a total of 92,558 individuals [36].

We fit a multiple linear regression model regressing R in 2015 against first year of publication, total number of publications by 2015, US-based or not, gender (male or female) and field of research. All variables were found to be significantly associated with R (p<0.0001 for all), although the magnitude of the coefficients tended to be small. The coefficient for total number of publications was -0.00150 per paper; the coefficient for being a US based author was 0.021 and the coefficient for being a male author was 0.028. Dividing these results by the standard deviation of R in 2015 for this dataset (0.35), yields a standardized effect size of -0.004 per paper for total publications, 0.06 for being US-based and 0.08 for being male. This indicates that, holding other variables constant, males and US-based authors have slightly higher values of R, although the effect is small.

### Correlation of R with citation impact (h-index)

Table 2 shows the association between R and h-index in unadjusted and adjusted analyses. Given that both h-index and R are continuous variables, we fit a multiple linear regression model between h and R, with the adjusted model controlling also for total number of publications, average number of co-authors, first year of publication, US-based or not, gender (male or female), all in 2015. These adjustments were pre-specified and they aim to remove the anticipated effect of these variables on h-index in probing the association between R and h. The results for each field are shown in Table 2 below. In most fields, a higher R (less collaboration) was associated with a significantly lower h-index (less citation impact). In the adjusted analyses, exceptions included biology, earth sciences, and social sciences.

**Table 2:** Linear regression effect of R on h-index in 2015, separated by field. Restricted to the 233,798 authors with N_p_ ≥ 30 and I_1_ ≥ 4 in 2015. The standardized effect is obtained by dividing the coefficient by the standard deviation of h in 2015 for each field. Results are shown unadjusted, and adjusted for total publications, average number of co-authors, first year of publication, US-based or not, and gender (male or female).

Field	Number of authors	Adjusted for other variables?	Coefficient	Standardized effect	p-value
Biology	14,839	Unadjusted	-1.07	-0.17	<0.0001
		Adjusted	0.24	0.039	0.27
Brain Sciences	11,594	Unadjusted	-1.64	-0.25	<0.0001
		Adjusted	-1.72	-0.26	<0.0001
Chemistry	25,065	Unadjusted	-0.45	-0.068	0.0066
		Adjusted	-0.59	-0.090	0.0062
Computer Science	37,532	Unadjusted	-1.18	-0.26	<0.0001
		Adjusted	-1.38	-0.30	<0.0001
Earth Sciences	5,043	Unadjusted	-1.59	-0.27	<0.0001
		Adjusted	-0.20	-0.034	0.56
Engineering	24,771	Unadjusted	-2.05	-0.37	<0.0001
		Adjusted	-2.81	-0.51	<0.0001
Health Sciences	12,372	Unadjusted	-1.31	-0.23	<0.0001
		Adjusted	-2.64	-0.47	<0.0001
Infectious Disease	7,617	Unadjusted	-1.34	-0.22	<0.0001
		Adjusted	-1.21	-0.20	0.00015
Medicine	54,326	Unadjusted	-2.05	-0.30	<0.0001
		Adjusted	-2.55	-0.38	<0.0001
Physics	35,922	Unadjusted	-12.37	-1.02	<0.0001
		Adjusted	-5.95	-0.49	<0.0001
Physics with R = 1.0 individuals removed	34,219	Unadjusted	-13.25	-1.09	<0.0001
		Adjusted	-5.69	-0.47	<0.0001
Social Sciences	4,717	Unadjusted	0.092	0.016	0.74
		Adjusted	-0.12	-0.022	0.73
*All fields*	233,798	Unadjusted	-7.44	-0.95	<0.0001
		Adjusted	-4.84	-0.62	<0.0001

### Principal investigator status and collaboration

Of the 400 US based authors randomly selected from brain sciences, infectious disease, and medicine, 135 were PIs in 2016 (33.8%). In our sample, PIs had higher citation h-index values than non-PIs (18.9 vs 15.5, p<0.0001, Student’s t-test). To determine the relationship between an author’s value of R in 2015 and whether they have achieved PI status by 2016, we fit a logistic model of PI status (a binomial variable) versus R, h-index, field, first year of publication, and gender, all for 2015. There was an association between higher R and being a PI by 2016, with an odds ratio of 2.84 (95% CI: 1.41 to 5.70, p = 0.0034) per 1 point change in R. The odds ratios for the association between PI and higher h-index was 1.08 (95% CI: 1.03 to 1.13, p = 0.0018) per 1 point change in h-index, for male gender 0.51 (0.30 to 0.85, p = 0.010), and for field of research being medicine 0.27 (0.14 to 0.53, p = 0.00014). There was no significant association between PI status and either first year of publication or total number of publications.

## Discussion

Collaboration is an essential part of scientific research, and different patterns of collaboration both shape scientific productivity as well as characterize individual scientific fields. In this work, we explored the use of an index for collaboration as measured by co-authorship patterns [1]. The R index captures the networking and co-authorship patterns of individual researchers, with lower values of R associated with researchers who more repeatedly work with the same groups of multiple co-authors on collaborative projects and higher values of R associated with more solitary investigators.

In this work, we showed that R varies widely across fields, with some fields displaying a considerably different pattern of collaboration and co-authorship than others. Results from 2006 to 2015 also show that R is a comparatively stable metric that effectively characterizes different scientific fields: R is relatively constant for most fields over this period. Some increases in R are seen in physics, chemistry, and medicine, but despite their nominal statistical significance (due to the very large sample size of authors analyzed), their effect size is very small. These findings are congruent with the results of previous studies of collaboration across scientific fields, such as Newman [38], which found different scientific fields to possess distinguishing network characteristics, including average number of collaborators per author.

In addition to the scientific field within which researchers work, a researcher’s gender and country may shape their pattern of collaboration. Above, we showed that males tended to have somewhat higher values of R than females, but the difference was small. This is similar to some previous results. For example, in one study of 36,211 Italian scientists, Abramo et al found that across scientific fields women have a slightly higher tendency to engage in collaboration, as measured by the fraction of publications resulting from collaboration [39].

Similarly, US-based researchers tended to have slightly higher values of R than researchers based in other countries, again indicating less overall collaboration. This may be because smaller countries tend to display higher rates of international research collaboration, that is, authors from smaller countries tend to collaborate more frequently with researchers from other countries [40]. In turn, larger countries such as the U.S. tend to offer more domestic opportunities for collaboration. Additionally, researchers from other countries may collaborate more frequently with researchers from the U.S., as these stable patterns of collaboration offer a way to increase the impact of their work. For U.S. based researchers, collaborating with researchers in other countries may not offer as much of an advantage, and as a result U.S. based researchers may collaborate slightly less.

Collaboration has previously been shown in several contexts to be associated with more cited, higher impact science [8]. To study this effect, we looked at the association between R and the h-index, and found that more collaborative researchers tend to have higher h indices. The direction of causality, if any, is uncertain. For example, it may be that higher impact researchers, through their greater visibility in their respective research fields, have an easier time finding opportunities to collaborate. Alternatively, it may be that researchers who tend to collaborate more frequently not only have more opportunities to co-author papers, but are also creating a larger network of citing scientists, thus increasing their overall citation impact.

This effect is not uniform across scientific fields, however. Within biology, earth sciences, and social sciences, there is not a significant relationship between R and h-index in 2015. Additionally, the association is strongest for physicists. This particularly strong association makes sense given the growing number of large, high impact, intensely collaborative projects in experimental physics [41].

We also studied the relationship between R and the tendency for researchers to become principal investigators (PIs) on NIH funded projects. Although PIs in our sample tended to have higher h-index values than non-PIs, we found that increasing collaboration as measured by R was associated with decreasing tendency to become an NIH-funded PI. On one hand, PIs tend to continuously change collaborators as graduate students and post-doctoral fellows enter and exit the PI’s labs. Thus, although PIs in federally funded biomedical research in the USA are often highly productive, they do not often engage in large, stable collaborative networks and hence this may explain their higher values of R.

This work has several limitations. First, for most of the analyses in this study, only authors with a total publication count greater than or equal to 30 and an I_1_ greater than or equal to 4 were included. These authors therefore represent some of the more productive researchers, and the relationships observed may not apply to less productive researchers. With small numbers of papers, values of R may have substantial noise and may fluctuate with the publication of each new paper. Second, we used the h-index as a measure of citation impact, but obviously many other measures are possible to consider as well as different says to standardize these measures [42]. While collaborative behavior was found to be associated with higher h-index, this may no longer be true or may be even inversed, if one were to use citation indices that penalize for extensive co-authorship (e.g. hm index). Third, PI status uses a definition that depends on obtaining NIH grants, but other scientists may also be team or group leaders but not involved as PIs in NIH funding. Acknowledging these caveats, our analyses offer a perspective about the patterns and correlates of collaborative behavior across diverse scientific fields. Fourth, it is possible that due to problems in name disambiguation, some author records are not perfectly clean, e.g. the works of the same author may be split in two Scopus IDs or the more than one authors’ work may be merged in the same Scopus ID. However, these errors are likely to be very uncommon, as previously shown [43] and Scopus has continuously improved name disambiguation since then. Therefore, name disambiguation problems are unlikely to affect the results substantially.
